# Epitranscriptomics in parasitic protists: Role of RNA chemical modifications in posttranscriptional gene regulation

**DOI:** 10.1371/journal.ppat.1010972

**Published:** 2022-12-22

**Authors:** Cassandra Catacalos, Alexander Krohannon, Sahiti Somalraju, Kate D. Meyer, Sarath Chandra Janga, Kausik Chakrabarti

**Affiliations:** 1 Department of Biological Sciences, University of North Carolina at Charlotte, Charlotte, North Carolina, United States of America; 2 Department of BioHealth Informatics, School of Informatics and Computing, Indiana University Purdue University Indianapolis (IUPUI), Indianapolis, Indiana, United States of America; 3 Department of Biochemistry, Duke University School of Medicine, Durham, North Carolina, United States of America; Joan and Sanford I Weill Medical College of Cornell University, UNITED STATES

## Abstract

“Epitranscriptomics” is the new RNA code that represents an ensemble of posttranscriptional RNA chemical modifications, which can precisely coordinate gene expression and biological processes. There are several RNA base modifications, such as *N*^*6*^-methyladenosine (m^6^A), 5-methylcytosine (m5C), and pseudouridine (Ψ), etc. that play pivotal roles in fine-tuning gene expression in almost all eukaryotes and emerging evidences suggest that parasitic protists are no exception. In this review, we primarily focus on m^6^A, which is the most abundant epitranscriptomic mark and regulates numerous cellular processes, ranging from nuclear export, mRNA splicing, polyadenylation, stability, and translation. We highlight the universal features of spatiotemporal m^6^A RNA modifications in eukaryotic phylogeny, their homologs, and unique processes in 3 unicellular parasites—*Plasmodium* sp., *Toxoplasma* sp., and *Trypanosoma* sp. and some technological advances in this rapidly developing research area that can significantly improve our understandings of gene expression regulation in parasites.

## 1. Introduction

By definition, a parasite is an organism that lives on or in another organism and feeds on it, ideally without killing it, yet often reducing its quality of life while the parasite exists within the host. Many protists are parasites that must infect other organisms to survive and propagate and they are mostly unicellular. There are about 200,000 known unicellular protozoa and about 5% of them have adapted to a parasitic lifestyle [1]. These parasitic protists have acquired countless niches dispersed across eukaryotic phylogeny, but majorly belonging to 2 phyla, namely, apicomplexa and kinetoplastida. Members of these phyla, *Plasmodium*, *Toxoplasma*, *Eimeria*, *Sarcocystis*, *Cryptosporidium*, *Theileria*, *Babesia*, *Trypanosoma*, *Leishmania*, and *Trichomonas* are known to cause severe diseases. In this review, we will focus on 3 unicellular parasitic protozoa that are representative members of the above 2 phyla: *Plasmodium falciparum* and *Toxoplasma gondii*, the 2 model Apicomplexa and the kinetoplastid parasite *Trypanosoma brucei*. Emerging evidence suggests that posttranscriptional gene regulation is a key component of genetic control mechanisms in these unicellular parasites, particularly in the regulation of their virulence genes [2,3]. While posttranscriptional regulatory mechanisms are critical for expression of stage-specific virulence factor proteins, the mechanisms underlying these processes are yet to be fully uncovered in parasitic protists. To this end, we focus particularly on regulatory changes through intrinsic RNA modifications [4], an exciting and growing area of research. Epitranscriptomic modifications, particularly *N*^6^-methyladenosine (m^6^A), are reversible marks that provide a mechanism for the potential dynamic regulation of gene expression [5–8]. Nearly all aspects of mRNA processing and metabolism, such as 5′ capping, splicing, polyadenylation, stability, and translation are affected by m^6^A and other RNA modifications [5,9–11]. Here, we review the current status of research on RNA regulation in parasitic protists involving RNA modifications with an emphasis on m^6^A modification, which has emerged as most prevalent in mRNAs and noncoding RNAs, and discuss how new techniques for defining epitranscriptomic signatures can enhance our understanding of m^6^A and other RNA modifications.

## 2. Gene regulation in parasitic protists

Among all the parasitic protists identified so far, *Plasmodium falciparum* is highly pathogenic and the deadliest parasite causing malaria in humans [12]. The high mortality rate associated with falciparum malaria is due to its unique biology and ability to multiply incessantly in host cells by evading human immune responses. The malaria infection is initiated when the female *Anopheles* mosquito, carrying the *P*. *falciparum* parasite, takes its first blood meal, injecting sporozoites into the human, which very rapidly makes their way to the liver to differentiate in the hepatocytes to reproduce asexually and become merozoites. The parasites now re-enter the bloodstream to invade erythrocytes (red blood cells or RBCs) and begin the intraerythrocytic developmental cycle (IDC) also referred to as the blood stage. The life cycle of *P*. *falciparum* is characterized by an exogenous sexual phase in the invertebrates (insects such as mosquito species: *Anopheles stephensi*) and an endogenous asexual phase (schizogony) in the vertebrate host (e.g., human). *P*. *falciparum’s* IDC depends on the varying levels of protein abundance to maintain its pathogenesis inside of the human host [13]. The IDC has historically been linked to resulting in the highest abundance of severe malaria cases through its ability to evade host immunity and further invade surrounding organs upon vascular adhesion. The phenotype of infected red blood cells (iRBCs) changes throughout the IDC, presenting parasite-made proteins integrating on the outer membranes of RBCs. Following the discovery of global gene expression profiles in the asexual IDC of *P*. *falciparum* [14–17], numerous studies have shown that *P*. *falciparum* in human RBCs has a cyclic pattern of steady-state mRNA expression, with more than 75% of the genes achieving high abundance of mRNAs at only 1 time point of their 48-hour life cycle [18]. Several studies have suggested that posttranscriptional control mechanisms are major means of gene expression regulation in malaria parasites in both sexual and asexual stages [19–22]. While posttranscriptional regulatory mechanisms are critical for expression of stage-specific virulence factor proteins, the mechanisms underlying these processes are yet to be uncovered in this apicomplexan parasite.

Toxoplasmosis, which results from infection with the *Toxoplasma gondii* parasite, is one of the most common parasitic diseases preying on immunocompromised individuals fitting the mold of an opportunistic pathogen. This obligate intracellular protozoan can infect any warm-blooded mammal but has 2 modes of transmission within its encysted forms, either found in undercooked animal products as tissue cysts or released as oocysts by feline feces [23]. Once inside of the newly infected host, the parasites can now mature into the tachyzoite phase propagating through the host and making itself known [23]. It is not until they differentiate into bradyzoites when they enter the more immuno-quiescent state within tissue cysts and can circulate throughout the body, reaching essential organs, such as the brain, kidneys, intestine, and bladder, making its way to achieving the life-long infection [23] as the unfortunate fact stands that there are currently no cures available. Transcriptional studies in *T*. *gondii* were performed primarily on tachyzoites and bradyzoites, which revealed highly heterogeneous gene expression patterns, including the expression of the large family of surface antigen (SAG)-related sequence (SRS) proteins that are known to mediate *T*. *gondii* attachment to the host cell, and thus, trigger host immune responses [24,25]. Posttranscriptional modifications of RNA transcripts were also implicated in the developmental regulation in *T*. *gondii* [26], including widespread pseudouridylation type RNA modifications, which are known to increase the stability of RNA transcripts [27]. Translational control is also a key part of gene expression regulation for many stage-specific proteins in *T*. *gondii* [26].

*Trypanosoma brucei* is a species that belongs to an ancient group of protozoans in the Trypanosomatidae family and thought to be evolutionarily divergent comparatively to other species within this family that also includes *Leishmania* and *Trypanosoma cruzi* [28]. *T*. *brucei* falls under the kinetoplastid group of parasites and survives by shuttling back and forth from a human host, as a “bloodstream form,” and an insect host (via a tsetse fly), as a “procyclic form,” the 2 most proliferative forms of this parasite. During the life cycle inside of the human, it lives extracellularly, initially, creating inflammatory chaos within the circulatory system and lymph causing trypanosomiasis, or sleeping sickness [29]. The following stage of this disease results in meningo-encephalic if the parasite makes it past the blood–brain barrier that results in obvious neurological symptoms [29]. Interestingly, during each host, the parasite differentiates from a highly proliferative cell cycle, reproducing rapidly via syngamy induced by flagellar interaction, to transforming to a quiescent form which is thought to be regulated by quorum sensing [30]. In *T*. *brucei*, antigenic variation is manifested by sequential expression of immunologically distinct variant surface glycoproteins (VSGs), its major surface antigen [31,32]. *T*. *brucei* has a large *VSG* gene pool [33], but only *VSGs* in expression sites (within 2 kb from the telomere) can be monoallelically expressed [34,35]. VSG switching occurs in the bloodstream form by a mechanism that has not yet been fully uncovered. The posttranscriptional processing in this organism is quite distinct compared to evolutionarily neighboring protozoan families as they contain no introns and opts for trans-splicing to assemble mature, messenger RNAs. There has been evidence suggesting varying levels within the methylome of *Trypanosoma* spp. playing a role in the regulation of life stage differentiation; however, exuberant understanding still lacks within this organism’s epitranscriptome regulation [36].

## 3. RNA-mediated regulation of co-transcriptional RNA processing events

Co-transcriptional RNA processing events are critically important for the maturation of pre-mRNAs and noncoding RNAs. Particularly, mRNA maturation requires several posttranscriptionally regulated processing events, such as 5′ capping, splicing, 3′ poly (A) tail addition (polyadenylation) and mRNA decay [37,38], which in turn affect cytoplasmic transport and translation of mRNAs [39]. Unicellular protists that are parasitic in nature, such as malaria pathogen *Plasmodium* spp., enteric coccidian parasite *Toxoplasma* spp., and kinetoplastid parasites, Trypanosomes or Leishmania, have evolved with both generic and specialized RNA processing pathways that provide an extraordinary glimpse of early eukaryotic evolution and the basis of phylogenetic diversity in RNA metabolism. Importantly, all above parasites develop through different life stages predominantly in mammalian or insect vectors, which require timely expression of genes/proteins for their growth and metabolisms, most notably, their virulence-associated genes essential for parasite survival. Emerging evidence indicates a significant proportion of spatiotemporal gene regulation in parasitic protists is dependent on posttranscriptional regulation at the RNA level [16,40]. However, a critical barrier to understanding the link between posttranscriptional control of timely, stage-specific protein expression in parasitic protists, and disease outcome is the lack of comprehensive information on genome-wide influence of the RNA-intrinsic features in parasite biology. From a plethora of studies in protozoan and metazoan species, it is evident that 2 important “RNA-intrinsic” features, RNA secondary structures and base modifications (also known as “epitranscriptomic” modifications), play pivotal role in RNA metabolism during the processing and maturation of mRNAs and noncoding RNAs. With the advent of high-resolution, next-generation sequencing technologies, and innovative molecular methods, it is now possible to dissect the mechanisms of posttranscriptional control at unprecedented depth and define their relationship to RNA modifications. Most notable are the application of long read, RNA sequencing by Nanopore and PacBio that allows accurate characterization of transcript isoform identification, detection of RNA modifications, and can be applied for high-resolution analysis of parasite transcriptomes.

## 4. RNA base modifications

Within the past half century, a multitude of RNA modifications have been identified and contribute to posttranscriptional regulation of cellular RNAs. The addition of chemical modifications presents an additional layer of regulation of all major classes of RNA. Highly abundant species such as rRNA and tRNA have been known for decades to be heavily modified, but it has not been until recently that cellular mRNAs have been shown to be susceptible to chemical modification. However, it is now recognized that mRNAs contain a diverse and dynamic “epitranscriptome” characterized by a growing list of chemical modifications present at varying abundances [41,42].

**Fig 1:**
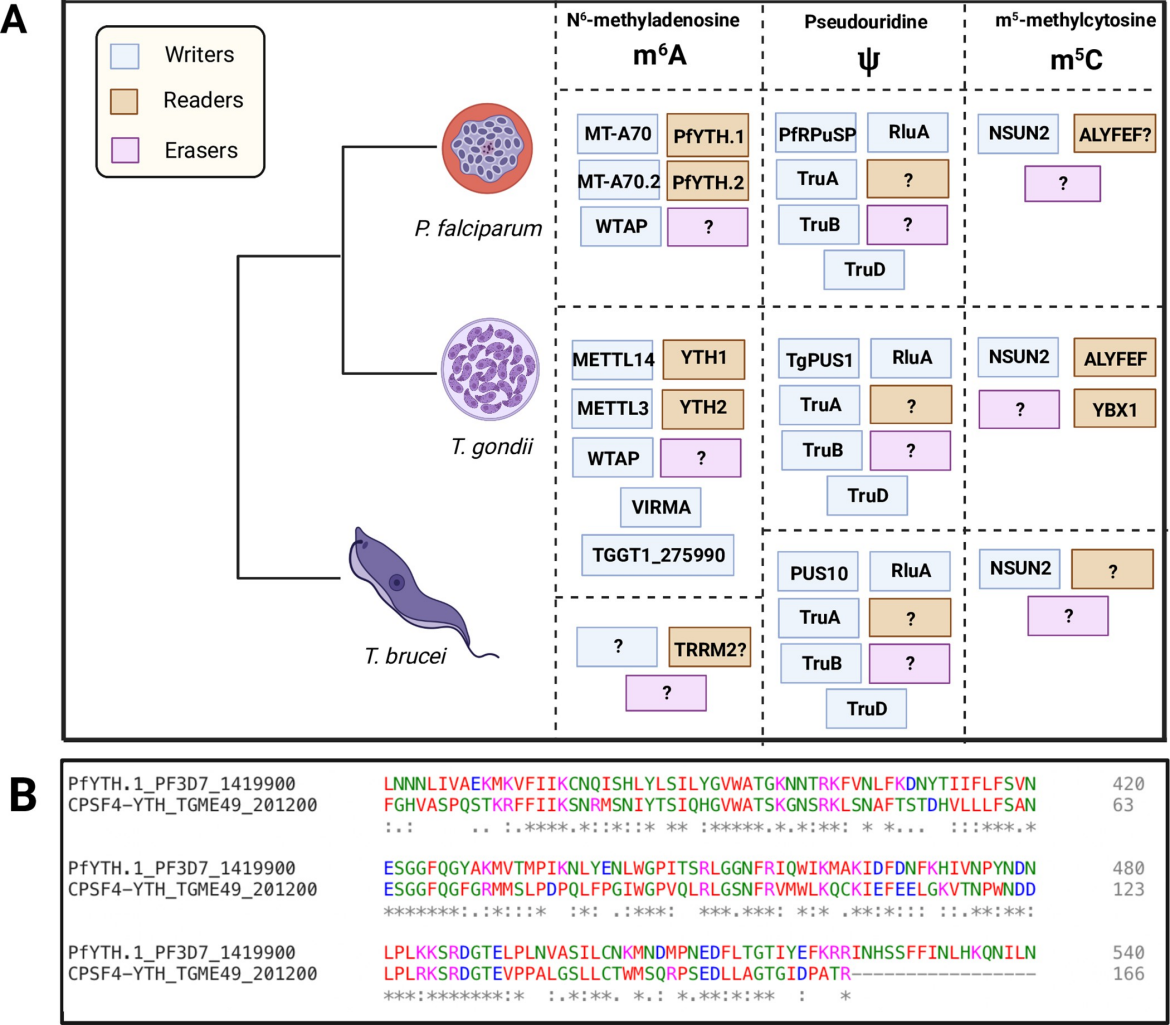
**(A) Protein orthologs that write, read, or erase RNA modifications in various branches of parasitic protists.** *P*. *falciparum*: ***N***^***6***^**-methyladenosine: m**^**6**^**A** (Writers) PfMT-A70 [70], PfMT-A70.2 [70], WTAP [70]; (Readers) PfYTH.1 [114], PfYTH.2 [114], CPSF-YTH.1 [94]; (Erasers) Unknown. **Pseudouridine: Ψ** (Writers) PfRPuSP [180], RluA [180], TruA [180], TruB [180], TruD [180]; (Readers) Unknown; (Erasers) Unknown. **M**^***5***^**-methylcytosine: m**^**5**^**C** (Writers) NSUN2 [125]; (Readers) ALYFEF? [125]; (Erasers) Unknown. *T*. *gondii*: **N**^***6***^**-methyladenosine: m**^**6**^**A** (Writers) METTL3 [71], METTL14 [71], WTAP [71], VIRMA [71], TGGT1_275990 [71]; (Readers) YTH1 [71], YTH2 [71], CPSF4-YTH1 [94]; (Erasers) Unknown. **Pseudouridine: Ψ** (Writers) TgPUS1 [181], RluA [181], TruA [181], TruB [181], TruD [181]; (Readers) Unknown; (Erasers) Unknown. **M**^***5***^**-methylcytosine: m**^**5**^**C** (Writers) NSUN2 [124]; (Readers) ALYFEF [124], YBX1 [124]; (Erasers) Unknown. *T*. *brucei*: **N**^***6***^**-methyladenosine: m**^**6**^**A** (Writers) Unknown; (Readers) TRRM2? [36]; (Erasers) Unknown. **Pseudouridine: Ψ** (Writers) PUS10 [182], RluA [182], TruA [182], TruB [182], TruD [182]; (Readers) Unknown; (Erasers) Unknown. **M**^***5***^**-methylcytosine: m**^**5**^**C** (Writers) NSUN2 [183]; (Readers) Unknown; (Erasers) Unknown. Created with BioRender.com. **(B)** Multiple sequence alignment (Clustal Omega) of PfYTH.1 (PF3D7_1419900) and CPSF4-YTH (TGME49_201200) protein showing conservation in the YTH domain of the C-terminus.

Of the mRNA modifications that have been identified in mRNAs, m^6^A has been characterized as the most abundant internal mRNA modification in almost all living organisms. m^6^A was first detected in animal mRNAs [43,44] and later found in viruses [45], bacteria [46], plants, and thousands of cellular RNAs, including both coding and noncoding transcripts [36,44,47,48]. m^6^A is a reversible modification that is deposited by the METTL3–METTL14 methyltransferase complex and removed by demethylases FTO and ALKBH5 (Fig 1A). In addition, a variety of RNA-binding proteins act as m^6^A “readers” by preferentially binding to m^6^A. Among these proteins are the YT521-B homology (YTH) domain family proteins, which contain a highly conserved YTH domain that specifically recognizes m^6^A. Several RNA processing events are impacted by m^6^A, including miRNA biogenesis, pre-mRNA splicing, polyadenylation, cellular localization, mRNA stability, and translation [49], and reader proteins are the primary mediators of these diverse effects [36,50]. In addition, m^6^A-dependent changes in RNA structure can influence gene expression through the control of translation or through impacting RNA:protein interactions [51].

This distinct chemical modification transforms the bioenergetics regarding RNA base pairing [52,53]. It has been reported that m^6^A can dynamically alter RNA structure [53]. Although not found to entirely deter m^6^A-U Watson–Crick base pairing through 2D NMR studies, the methylamino group energetically favors *syn* geometry, which places m^6^A in the major grove in the context of double-stranded RNA, destabilizing duplex structures [54]. Single-stranded RNA can be energetically favored through stronger associated base stacking in unpaired, methylated conditions and adjacent bases [54]. However, m^6^A has also been reported to stabilize m^6^A-U pairing when neighboring a 5′ bulge, indicating that it is not universally disruptive to RNA structures [53].

**Box 1**

Oxford Nanopore Technologies (ONT) has arisen as a third/fourth generation sequencing technology capable of directly sequencing DNA and RNA samples without the need of cDNA conversion. Recently, direct RNA sequencing using ONT sequencers have been used to detect m^6^A and other RNA modifications [55,56]. In traditional ONT analyses, total RNA is extracted from the biological sample. Poly(A) mRNA are then enriched from the sample, typically through the use of microbeads or other chromatographic methods [57,58]. A corresponding cDNA is often synthesized to prevent the RNA from forming secondary structures and to protect against degradation from RNases. An RNA sequencing adaptor (RMX), carrying a “motor protein” is ligated to the 3′ end of the RNA, while a “tether” protein is ligated to the 5′ end of the cDNA.

The extracted cDNA molecules are injected onto a flow cell channel connected to an ONT reader device. The flow cell contains hundreds of small nanopore proteins (green), with a nearly 1 nm aperture, embedded in an electro-resistant membrane (black). When the motor protein (yellow) on the cDNA (blue) comes into contact with the nanopore, the difference pulls the connected RNA (red) through the pore, while the tether protein (violet) blocks the cDNA from entering. As the molecule traverses the roughly 5 nucleotide length channel, it disrupts the electrical differential of the membrane. This disruption is captured by a small electrode near the base of the pore and transmits the change in current to the receiver in the sequencing device. This process continues until the RNA has completely passed through the pore, then the motor protein dissociates, allowing another motor protein to attach and begin the process anew.

The current recorded by the electrode is recorded as a squiggle. Each change in potential is characteristic of a particular 5-mer, the 5 nucleotides contained in the pore, and can be analyzed using machine learning models. This process of reading a squiggle to predict the nucleotide sequence is called base-calling. After base-calling, the resulting long-reads can be used for numerous downstream analyses: de novo genome assembly, transcript isoform discovery, differential gene/transcript expression analysis, or mapping nucleotide modifications at single nucleotide and single molecule resolution [59–61].

Since the development of nanopore sequencing, numerous different base-callers have been created, not only to predict the 4 constituent bases, but also to predict specific base modifications. This is possible due to the distinctly different change in current caused by a 5-mer containing a modified base, relative to a 5-mer with an unmodified base. The characteristic difference in current due to RNA base modifications results in base-call errors in those regions, leading to a preponderance of base-call errors at loci encoding for modified bases [62]. Since ONT platform does not require PCR amplification steps, they are supposed to exclude the sequencing or base-calling bias that may arise from GC- or AT-rich genomes [63], such as in *P*. *falciparum* (approximately 90% AT-richness in the intergenic regions).

### 4.1 Writers/readers/erasers in parasitic protists

#### 4.1.1 Writers

m^6^A is deposited by the METTL3 methyltransferase enzyme, which interacts with a complex of accessory proteins including METTL14, WTAP, VIRMA, RBM15/15B, ZC3H13, and HAKAI that function to guide METTL3 to target RNAs and promote the stability and localization of the complex to nuclear speckles [64,65]. In mammals, a megadalton protein complex is responsible for deposition of the m^6^A mark, which includes methyltransferase-like protein 3 (METTL3) [66]. This active adenosine methyltransferase catalyzes a methyl group in its co-factor S-adenosyl-L-methionine (SAM) and transfers to N6 atom of adenine in RNA [67] The methyltransferase complex directs methylation in a DRACH (D = Guanosine/Adenosine/Uridine; R = Guanidine/Adenosine; H = Adenosine/Cytidine/Uridine) consensus motif, and in mammalian cells, the methylation is enriched in proximal 3′ UTRs and long internal exons [48,68]. In addition, a number of regulatory subunits are involved in mammals, including Wilms’ tumor 1-associating protein (WTAP) that stabilizes the interaction between METTL3 and METTL14 [69].

m^6^A has recently been uncovered as an abundant modification in *P*. *falciparum*, relative to its approximately 85% AT-rich genome [70]. In *P*. *falciparum*, the annotation differs, but the main writer proteins, WTAP, MT-A70, and MT-A70.2, are homologous to the evolutionarily conserved RNA methyltransferase core proteins found in other eukaryotes. In *P*. *falciparum*, the mammalian ortholog of METTL3 has been identified and designated as PfMT-A70 (PF3D7_0729500) [70]. The writer complex in this organism has not been characterized as well as in other protozoan species like *Toxoplasma*, in which the additional proteins VIRMA and TGGTI_275990 have been identified [71]. HHPHRED webservers have made strong homology associations between WTAP and TGGTI_275990 although it remains unannotated [71,72].

In Trypanosomatids, direct orthologs of the methyltransferase writer complex have not been identified [73]. However, 50% of total cellular m^6^A appears in essential genes encoding for VSGs in the *T*. *brucei* bloodstream form proliferative stage, particularly in the poly(A) tail [36,74]. It was shown that in the absence of m^6^A in the 3′ UTRs of VSGs, accelerated deadenylation occurred leading to the degradation of mRNA [74]. A conserved upstream 16-mer motif (5′-TGATATATTTTAACAC-3′) in the 3′ UTR of VSGs has been identified as necessary for m^6^A incorporation in the poly(A) tail [74].

#### 4.1.2 Readers

YTH domain-containing proteins are the most well-characterized m^6^A reader proteins. These proteins contain a highly conserved YTH (YT521-B homology) domain that is responsible for m^6^A recognition. Humans possess 5 YTH domain-containing proteins either characterized as YT521-B homology (YTH) domain family proteins (YTHDF) or as YT521-B homology (YTH) domain-containing proteins (YTHDC). Mammalian YTHDF and YTHDC proteins, *Arabidopsis* CPSF30, budding yeast Pho92, and fission yeast Mmi1 contain this m^6^A reader domain, although the Mmi1 protein does not preferentially bind m^6^A [185]. YTHDC1 localizes to the nucleus and plays important roles in pre-mRNA splicing, noncoding RNA function, and epigenetic silencing and activation [75–79]. YTHDC1 is important for germ cell development and contributes to mRNA abundance and translation regulation [51,80,81]. The “DF” proteins YTHDF1, 2, and 3 are a major family of cytoplasmic m^6^A readers and have been implicated in the regulation of mRNA stability, translation, and localization [49,82–85]. A longstanding model for these proteins has been that they have unique functions, with DF2 primarily promoting mRNA degradation, DF1 promoting translation, and DF3 contributing to both degradation and translation. Recently, however, this model has been challenged by studies demonstrating functional redundancy among the DF proteins, with all 3 contributing to mRNA decay [86–88]. Interestingly, our group recently discovered that individual mRNAs can be bound by multiple DF proteins throughout their lifetime, suggesting that DF proteins may not immediately act to degrade their target mRNAs [89]. Further studies examining the roles of DF proteins in mRNA regulation in distinct cell states and across unique cell types will likely provide more insights into how these proteins function to control methylated mRNA fate.

Two orthologs of YTH domain-containing proteins have been identified in *P*. *falciparum*: PfYTH.1 (PF3D7_1419900) and PfYTH.2 (PF3D7_0309800) [90]. In *Plasmodium* species, the PfYTH.2 protein contains an aromatic amino acid cage that forms the methyl-binding pocket. More specifically, the phenylalanine in the 98th position plays a significant role in successful m^6^A binding to PfYTH2 [91]. Structural analysis of YTH domain complexes in mammalian systems reveal similar residues required for m^6^A recognition and affinity. YTHDC1 contains hydrophobic amino acids, W377, W428, and L439 that act as an aromatic cage that is stabilized through the methyl-*π* interaction [92]. The adjacent cytosine has also found to play a crucial role in reinforcing the YTHDC1-m^6^A complex through the cation- *π* of the C+1 and positively charged R475 on YTHDC1 [92]. HsYTHDF1 has even greater homology to PfPfYTH proteins [70,93] and contains the necessary aromatic residues to recognize m^6^A, W411, W465, and W470 [92].

Interesting comparisons between metazoan and protozoan species distinguishing not only the m^6^A methyltransferase complex, but readers recognizing m^6^A on the 3′ end of the transcript, have brought rise to investigating alternative lengths and instability mediated by Cleavage and Polyadenylation Specificity Factor-30 (CPSF-30) [94]. This aligns with the investigation of how m^6^A plays a role in determining the fate of transcripts, therefore, protein abundance, in some of the most pathogenic species within infectious disease, such as *P*. *falciparum* and *T*. *gondii* [95]. The architecture of *P*. *falciparum* protein, PfCPSF-30 (renamed to PfYTH.1) contains tandem zinc finger domains adjacent to a YTH domain. This plant-like polyadenylation specificity factor with an m^6^A reader domain provides a unique opportunity to target this protein family in parasites to develop antiparasitic drugs and thus avoid unwanted human interactions.

#### 4.1.3 Erasers

Two demethylase proteins (“erasers”) have been identified that can remove the methyl group from m^6^A. These include fat mass and obesity-associated protein (FTO) and ALKBH5, members of the AlkB family of proteins that are nonheme Fe (II) and α-ketoglutarate-dependent dioxygenases [96,97] FTO localizes primarily to the nucleus, although its subcellular distribution can vary by cell type, and in some cells, it can be detected in the cytoplasm [96]. An important discovery came when Mauer and colleagues found that FTO can also demethylate m6Am, the first nucleotide of the 5′ cap [98]. This challenged the notion that FTO is exclusively an m^6^A demethylase, and indeed, subsequent studies found that FTO acts on m^6^A, m^6^Am [99]. ALKBH5 was the second m^6^A demethylase to be discovered, and unlike FTO, it does not have activity toward m^6^A_m_ [97]. A murine study showed a lack of ALKBH5 correlates with male infertility from inappropriate splicing that would have been facilitated by m^6^A in key positions resulting in an accumulation of shorter transcripts [100]. The idea that m^6^A can be reversed in cells suggests the possibility for dynamic regulation of m^6^A. However, these proteins do not appear to act globally on all m^6^A residues, but rather can remove m^6^A from specific transcripts in distinct cell types or cellular environments. Moreover, m^6^A profiling in chromatin-associated and nucleoplasmic mRNA has shown that the majority of m^6^A sites are conserved in cytoplasmic mRNA, arguing against the idea of widespread demethylation [101]. Further work is needed to better understand the factors that determine which transcripts and which m^6^A sites are targeted by FTO and ALKBH5 in different cell types.

Notably, there have not been homologous eraser proteins detected in *Plasmodium* or *Toxoplasma* species and proteins such as FTO remain exclusively found in the vertebrate lineage. One reason for this could be unicellular, protozoan organisms advance through their life cycles requiring differentiation for transmission and overall survival. As a result, there are noticeable, routine phenotype changes. With ambitious requirements from such small genomes, there would be no reason to eliminate a modification that aligns with diversifying the range of expression these organisms can obtain. However, ALKB family protein, TbABH, has been identified in *T*. *brucei* that showed amino acid identity with known FTO [102].

### 4.2 Heterogenous ribonucleoproteins (HnRNPs) and other players

Although YTH domain-containing proteins contain the highest affinity for binding to m^6^A, alternative proteins can directly bind to this modified base that allows them to fall under the “reader” category. A translation initiation factor, eIF3 [10], has been identified, IGF2BP [103] proteins, FMR1 [104], and a handful of heterogeneous nuclear ribonucleoproteins (hnRNPs). HnRNPs are multidimensional in their involvement in the regulation in the cell resulting from their interaction with RNA during mRNA processing events. The requirement of Arg-Gly-Gly repeats lends itself the ability to directly bind to a purine-rich region overlapping with the m^6^A motif [105]. It has been found that RNA structure changes induced by m^6^A additions provide recognition of RNA binding motifs for hnRNP-C to facilitate alternative splicing at the target mRNA [106], which is known as the “m^6^A switch.” In the absence of m^6^A, RNA hairpins bury the U-rich stretch that inhibits recognition [107]. The m^6^A-mediated increased accessibility of RNA leading to alternative splicing has also been recognized by hnRNP-G. HnRNP-A2B1 elicits similar alternative splicing and processing roles, however, has been found predominantly interacting with microRNA facilitation [108].

No hnRNP orthologs have been identified in *Plasmodium* or *Toxoplasma* spp. However, hnRNP F/H homologs have been found in *Trypanosoma* through proteomic analysis that is differentially expressed throughout their life cycle being more ubiquitous during the human bloodstream form parasite [109]. Within its genome, purine-rich motifs flank 3′ splice sites and polyadenylation sites of genes found to be regulated by this protein.

## 5. Epitranscriptomics of mRNA and noncoding RNA processing in posttranscriptional regulation in parasites

### 5.1 5′- capping

The co-transcriptional 5′ 7-methylguanosine cap is required for mRNA stability and translation initiation while the transcript enters the cytoplasmic environment. There are known events that take place in the 5′- UTRs of certain viral strains that do not require a 5′ cap when large structural elements are present, known as internal ribosomal entry sites (IRESs). Eukaryotic organisms require a high level of complexity at the 5′ ends to safely exit the nucleus, proceed with protein translation, and be identified as endogenous. *N*^6^,2′-O-dimethyladenosine (m^6^A_m_) is an extension of the 5′ cap that can occur on the first transcribed nucleotide if it is an adenosine. This addition is made by the PCIF protein [110]. The addition of m^6^A_m_ has been shown to impact both mRNA translation and stability [110,111].

Although most translation is achieved through recognition of the 5′ cap, the m^6^A modification has been shown to facilitate cap-independent translation under some conditions [10,112]. For example, 5′ UTR m^6^A can recruit the translation initiation factor eIF3 to select mRNAs and facilitate cap-independent translation during stress [10]. Additionally, in response to heat shock, YTHDF2 translocates to the nucleus and binds to m^6^A sites within the 5′ UTR of *Hsp70*, protecting it from demethylation of FTO [113]. Although this mechanism has proven true in mammalian cell lines, there is currently not enough evidence to suggest m^6^A mediated 5′ cap-independent translation initiation contributes to the parasite translation apparatus. During interactive proteomic assays, researchers reported elongation initiation factor 3 (eIF-3) existing in the same complex as PfYTH.2, presenting possibility for this avenue in *Plasmodium* and permitting further exploration related to this mechanism [114]. Although this interaction may suggest a similar mechanism, there are no known m^6^A demethylase enzymes to require protection under similar conditions.

### 5.2 Polyadenylation and mRNA stability

Most eukaryotic mRNA precursors (pre-mRNAs) must undergo extensive processing, including cleavage and polyadenylation at the 3′-end. Particularly, 3′-end processing promotes mRNA stability and translation. Processing at the 3′-end is controlled by sequence elements in the pre-mRNA (*cis* elements) as well as protein factors. The 3′-end cleavage and polyadenylation reaction is directed by sequence elements within the untranslated region (UTR) of the pre-mRNA (the so-called *cis* elements), which includes an “Adenosine”-rich, hexameric AAUAAA polyadenylation signal (PAS). This molecular process is located at the 3′ end of the newly RNA Pol(II) synthesized RNA that traditionally requires the polyadenylation polymerase to make the addition of a poly(A) tail, accompanied by a poly(A)-binding protein, once cleavage occurs 25 to 30 nucleotides downstream from the polyadenylation signal motif [115].

Interestingly, in plants, adenosines within the multi-partite plant polyadenylation signal could themselves be methylated, raising the possibility that recognition of m^6^A marks by the plant polyadenylation protein, CPSF30 [116], may contribute to mRNA polyadenylation. *Arabidopsis* mutants deficient in the form of CPSF30 that “reads” m^6^A marks show genome-wide alterations in poly(A) site choice, consistent with this possibility. This evidence suggests an intimate link between polyadenylation machinery and the epitranscriptome in the mechanism of 3′ end processing in eukaryotes. Similarly, to plants, YTH-family reader proteins constitute part of the machinery involved in processing the cleavage and polyadenylation in apicomplexan parasites, such as *Toxoplasma* and *Sarcocystis* species [117,118].

A YTH domain has been discovered to be contained within CPSF30 (also seen as CPSF4-YTH) in *Arabidopsis* suggesting the polyadenylation pathway’s involvement with m^6^A in the 3′ end of the transcript [116]. Once that discovery was made, the further exploration of similar homology was sought out for in other species concluding the following organisms containing this homology: *Plasmodium falciparum*, *Babesia bovis*, *Eimeria tenella*, *Toxoplasma gondii*, *Neospora caninum*, *Chromera velia*, *Vitrella brassicaformis*, and *Arabidopsis thaliana* [94]. Organisms not containing YTH domain homology in poly(A) proteins include: *Theileria parva*, *Cryptosporidium parvum*, *Perkinsus marinus*, *Tetrahymena thermophila*, *Chlamydomonas reinhardtii*, *Saccharomyces cerevisiae*, *Schizosaccharomyces pombe*, *Caenorhabditis elegans*, *Drosophila melanogaster*, and *Homo sapiens* [94]. In fact, the lack of recognition when m^6^A is nonexistent along key sites in the 3′ untranslated regions created chimeric mRNA events, potentially offering an alternative isoform opportunity through polyadenylation (polyA) site choice, adding yet another tool in this organism’s arsenal to expand on its minimal genome requirement. Alternatively, the polyadenylation signal (PAS), AAUAAA, being A-rich, in addition to intergenic poly(A) stretches, offers an opportunity for m^6^A to interact with RBPs that are recruited to these locations. With the disproportion of adenines along the 3′ ends of *P*. *falciparum* transcripts, there is possibility of erroneous cleavage leading to the truncation of proteins in addition to leaving mature mRNA without a 3′ UTR. As seen in Fig 1B, the C-terminus is highly conserved between TgCPSF4-YTH and PfYTH.1, indicating the possibility of similar function in *P*. *falciparum* that exists in *T*. *gondii*. Rather than allowing for cellular damage, protozoans may have adapted to find utilization in chemical modifications, especially m^6^A, to act as a supplemental signal to benefit by their A-rich nature.

Important regulatory sequences are housed in the 3′ end of mRNAs, such as microRNA and long noncoding RNA complementary sites. As RNA silencing pathways do not exist in some of the unicellular eukaryotes (such as *Plasmodium* spp.), mammalian cells could use this modification to overthrow its degradation mediated by interfering RNA. Although there is no evidence suggesting homology between CPSF30-YTH, in *Plasmodium* (PfYTH.1) and *Toxoplasma*, and proteins involved in polyadenylation in humans, HsYTHDF1 appears to show consistent YTH containing C-terminal domains with a flexible N-terminus [70,93,119]. The shared flexible low-complexity region (LCR) offers potential for compositional plasticity presenting insight to binding capabilities on structural targets, such as transient RNA secondary structures [120]. Additionally, a group discovered m^6^A associated methyltransferase protein, VIRMA [121]. It was found that this protein is a fundamental part of recruiting the basic methyltransferase catalytic core, primarily adding m^6^A in the 3′ UTR and near the stop codon facilitating alternative polyadenylation in *Toxoplama* spp. [121]. An experiment performed on HeLa cells provided insight that in the event of a VIRMA knockout, there is a result of 3′ UTR extension, 84% of which are typically m^6^A enriched [121]. In fact, VIRMA showed a substantial association with polyadenylation cleavage factor CPSF5, furthermore, suggesting m^6^A mediated polyadenylation occurring in mammals [121]. The significant overlap of m^6^A signatures with the *cis*-acting elements of the polyadenylation process indicates there is potentially important molecular choreography between m^6^A modifications in addition to the polyadenylation machinery being a key process in maturation of mRNAs in parasitic protists. Compelling experiments performed by independent groups further explored m^6^A’s importance in the viability of transcripts facilitated by appropriate 3′ end processing in *T*. *gondii* [71,94]. A knockdown study of proteins paramount for m^6^A installment (METTL3 and WTAP) resulted in a complete arrest of parasite replication and impairs appropriate 3′ end formation, which makes perfect sense being that m^6^A distribution along the transcriptome was primarily found to reside near 3′ transcript ends [71]. A group later looked at this mechanism in a way that was previously analyzed as plant-like, in *Arabidopsis thaliana*, being that the shared YTH domain is conserved in polyadenylation specificity factors (CPSF4) in both *A*. *thaliana* and *T*. *gondii* [94]. Using direct RNA sequencing, they provided evidence of chimeric, transcript read-throughs in stage-specific genes in the absence of CPSF4 or m^6^A enrichment via METTL3 knockdown [94].

In mammals, mRNA stability is intimately linked to m^6^A and other RNA modifications. Previous studies have found that m^6^A can negatively regulate mRNA stability [82,122]. Corroborating studies on m^6^A-binding proteins suggest that the knockdown of YTHDF reader proteins can increase the mRNA stability since reader proteins, such as YTHDF1, YTHDF2, and YTHDF3, can reduce the stability of m^6^A containing mRNA leading to its degradation [82,123]. This suggests a direct link between mRNA polyadenylation and the epitranscriptome in maintaining mRNA stability. In addition to m^6^A, other RNA modifications, such as m^6^A_m_, pseudouridine (Ψ), and m^5^C can significantly affect eukaryotic mRNA stability [124]. Notably, in parasitic protist *P*. *falciparum*, m^5^C affects mRNA stability and contributes to its sexual stage development [125].

Trypanosomes contain a unique feature being the poly(A) tail itself has been found to contain m^6^A. Initially, a study found mRNA enrichment following a MeRIP experiment in procyclic form (355) versus bloodstream form (95) suggesting m^6^A takes a stage specific role [36]. This group also found longer half-lives associated with m^6^A containing transcripts [36]. Further validation later came independently from another group who revealed m^6^A being located in the poly(A) tail with a 16-mer motif located upstream in the 3′ UTR [74]. The link between m^6^A in the 3′ end and degradation was addressed in the context of VSGs transcripts [74]. They observed 50% of global m^6^As are located within the poly(A) tail of VSGs and are crucial for transcript stability [74]. Removal of this modification resulted in deadenylation and transcript degradation [74].

### 5.3 Noncoding RNA (ncRNA) processing

Like protein coding RNAs, noncoding RNAs (ncRNAs) undergo extensive processing. In humans, the coding RNA accounts for approximately 1% to 2%, while in protists, the number slightly increases to around 3% to 4%; however, the great majority of RNA species remains noncoding, making noncoding RNA’s importance quite clear in cellular processes. NcRNAs are generally classified as small RNAs that are <200 nucleotides in length or long RNAs that are >200 nucleotides long. Potential for how the cell utilizes modified ncRNA opens the gates for further exploration in all organisms once the hurdle of characterization has been accommodated.

Recently, METTL4 was found to take on a novel catalyzing role and in addition to its m^6^A_m_ addition involving splicing, reports highlight the addition of m^6^A on microRNA [126]. Long noncoding RNAs (lncRNAs) have been proven to play a significant role in the regulation of the cell cycle in eukaryotes [36]. THOR is an lncRNA known for being a testis-associated oncogene [36]. In lncRNA THOR, induced mutations contributing to the absence of m^6^A within key functional stem loops resulted in a lack of stabilization and increased degradation suggesting an m^6^A dependent modality [36]. Mutations in key m^6^A sites have been found to cause cellular dysregulation resulting in cancer and disease. Additionally, dysfunctional m^6^A regulators (readers, writers, and erasers) contribute to tumorigenesis and cellular proliferation in multicellular organisms. Understanding the mechanistic regulation of m^6^A in cancer therapy response and resistance could indicate potential targets for precise therapeutics [127]. Circular RNAs (circRNAs) are generated from back-splicing and have been found to play a regulatory role in the cell; however, key identifiers are required to increase the diverse functions such as transcription regulation and templates for translation [128]. Additionally, they can act as RNA decoys for miRNA and RBPs to enhance posttranscriptional modulation [36,129]. m^6^A additions on circRNA can recruit eIF proteins, when the reader protein is utilized as an adaptor, further indicating translation involvement [127]. So far, very little is known regarding epitranscriptomic landscapes of lncRNAs and circRNAs in parasitic protists. However, a particular lncRNA in *T*. *brucei*, snoGRUMPY, was recently found to play a role in cell differentiation and is a C/D box snoRNA family [130]. The typical role of this family of snoRNA is guiding 2′-O-methylation (Nm) in rRNA, which was tested using RiboMeth-seq (ribose methylation sequencing) [130]. Remarkably, snoGRUMPY was found to increase translation efficiency by either directly binding to mRNA via antisense or by methylation of transcripts [130].

Long noncoding RNAs are known to play a crucial role in genome integrity, particularly for the maintenance of chromosome ends, known as telomeres [131]. One of the RNAs works in conjunction with proteins to form a ribonucleoprotein enzyme complex known as telomerase [131]. Telomerase RNA (TR) has been found to contain the m^6^A modification particularly in human TR (hTR) [124]. ALKBH5 regulates the activity of hTR using m^6^A removal that is consistent with the loss of trimethylguanosine synthase 1 (TGS1) resulting in a dysfunctional hTR from mislocalization [132]. Although the identity of TRs in parasitic protists *P*. *falciparum* [133] and *T*.*brucei* [134] are known, the m^6^A-TR interaction and their functional consequences remain uncharacterized. The other RNA, known as telomeric repeat-containing RNA or TERRA plays important roles in telomere biology, including regulation of telomerase activity and heterochromatin formation at chromosome ends. This G-rich RNA is known to form G-quadruplexes (G4) that contain stacked Hoogsteen-bonded G-quartet motifs stabilized by monovalent cations [135]. Recently, it was found that the methyltransferases that are involved in epitranscriptomic function show a binding preference for RNA G-quadruplex (rG4) structures in TERRA RNAs, via the METTL14 RGG repeats [136]. Although TERRA RNAs are identified in some parasitic protist, such as *T*.*brucei* [137], m^6^A abundance in these 2 RNAs and their specific role in unicellular parasitic protists remains unexplored.

Transfer RNAs (tRNAs) are required for the synthesis of proteins as they are the adaptor molecule between the messenger RNA and the growing string of amino acids. Just like other eukaryotes, tRNAs in protozoan organisms are heavily modified. In *P*. *falciparum*, emerging evidence suggests tRNAs behave differently depending on the presence of particular RNA modifications in key structural elements [138]. In *P*. *falciparum*, following tRNA modifications exist: ncm^5^U, mcm^5^U, mcm^5^s^2^U, ψ, s^2^U, Gm, Cm, I, and m^5^C [138]. An elegant set of experiments in *P*. *falciparum* defined dynamic tRNA modifications as the parasite undergoes 3 stage-specific “reprogramming” events highlighted by the up-regulation of Am and 2-thiouridine modifications in the ring stage, an elevated level of a set of different methyl modifications that happened during the ring-to-trophozoite transition period and the dynamic changes affecting specific wobble tRNA modifications in the late-stage parasites. These “reprogramming” events are coupled to codon-biased protein expression system to fine-tune parasite gene expression across IDC. In this parasite, m^6^A is not found to disrupt Watson–Crick base pairing, therefore, will not contribute to wobble modification facilitated translational modulation [138]. PfDNMT2, which has previously been proposed as exclusively a DNA methylation enzyme, has shown to take a pivotal role in tRNA^Asp(GTC)^ regulation through cytosine methylation at the 38th nucleotide position [139]. A PfDNMT2 knockout study revealed this modification position plays a role in cellular stress response by perturbing codon-bias proteins crucial for stage-specific homeostasis [139]; 2-thiourea (s^2^U) has proven importance in all living organisms contributing to aminoacylation, structural stability, and the recognition of codons [140]. Before the recent study by Yang and colleagues, it was not certain whether *T*. *gondii* contained a tRNA thiouridylase enzyme, TgMnmA [140]. This group found that knocking out *Tg*MnmA led to abnormalities in apicoplast biogenesis [140] and because of its conservation in all apicomplexans, this apicoplast-specific tRNA- enzyme is considered as a potential drug target.

## 6. Epitranscriptomics of translational regulation

m^6^A and other RNA modifications have emerged as important regulators of cellular translation [4,8,51,84,119]. A recent study on comprehensive characterization of m^6^A modifications in *P*. *falciparum* coding regions over the course of blood-stage development showed that m^6^A is highly developmentally regulated in *P*. *falciparum*, and m^6^A levels is relatively higher than any known eukaryotic species [70]. Importantly, more than 99% of m^6^A sequencing reads mapped to protein-coding regions; however, there was a negative correlation of m^6^A RNA modification with translation efficiency, suggesting that CDS m^6^A is likely enriched in transcripts with inactive translation in *P*. *falciparum*.

**Fig 2:**
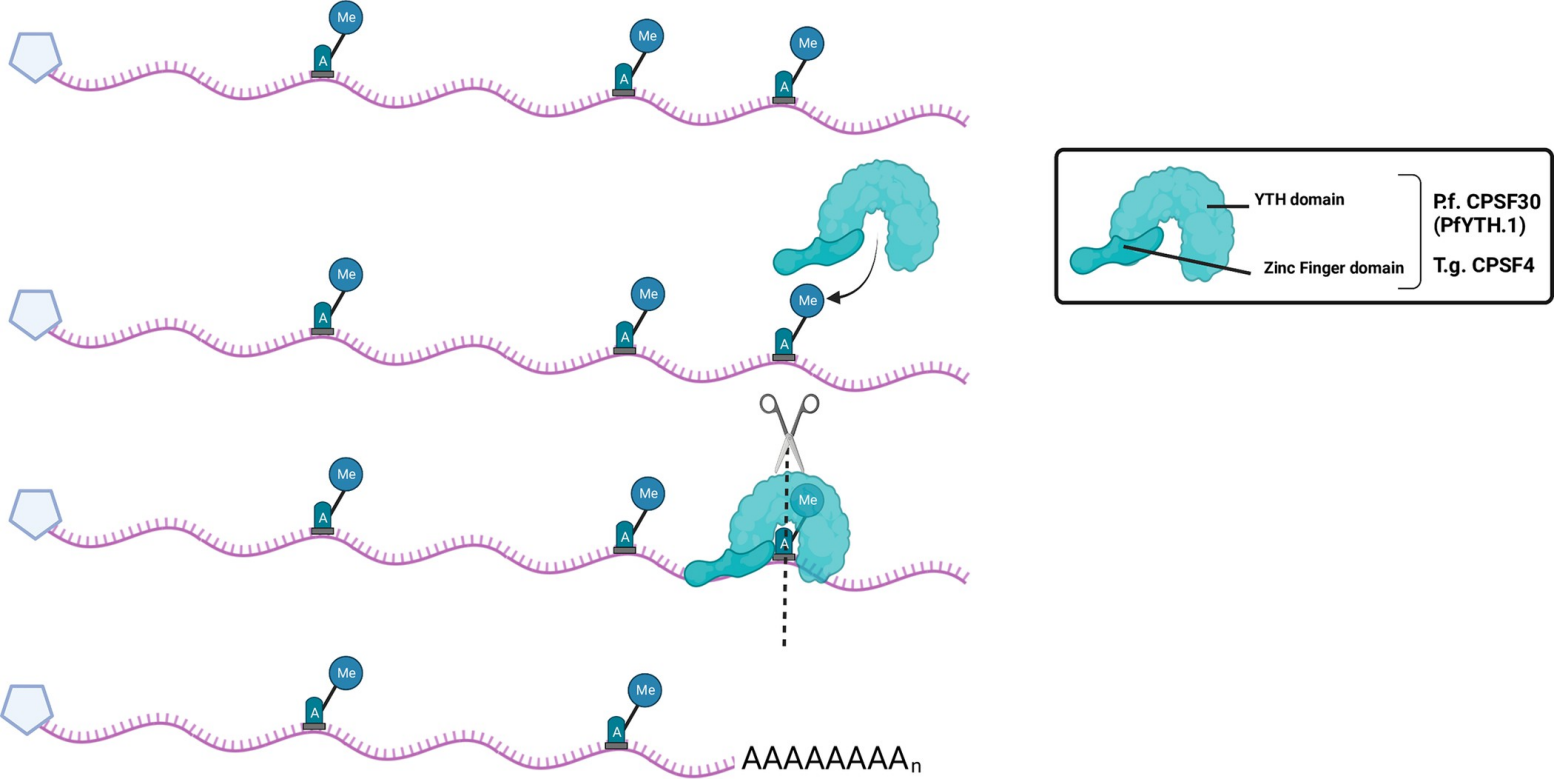
Model demonstrating m^6^A facilitated transcript end site recognition mediated by YTH containing protein, CPSF4 in *T*. *gondii* [71,94]. As stated in literature, where the m^6^A is installed, polyadenylation machinery may recognize the location and carry-out recruitment of associated proteins, cleavage (dotted line), and poly(A) tail addition. Created with BioRender.com.

Ribosomal RNA (rRNA) plays a crucial role in the ribosomal machinery necessary for protein translation [141]. Fully mature rRNA contains modifications helping ribosomes achieve its native conformation and active form [142]. This housekeeping structural RNA behaves uniquely to its folded structure and can be heterogeneous depending on the location of the chemical modification [142]. It has been noted that m^6^A helps to reinforce the rRNA structure; however, its addition is not made by the conventional mRNA catalyzing methyltransferase [70]. ZCCHC4 behaves catalytically as a novel methyltransferase that imprints m^6^A particularly on the 28S rRNA [70]. Ribosomal RNA in parasitic organisms, such as *Plasmodium*, are found to be heavily modified, with most known 2′-O-ribose methylation and pseudouridine [133,143], especially to provide stability in rRNAs to achieve its functional confirmation [144]. Specifically, rRNA adenine dimethyltransferase (rAD) has been well characterized in its ability to dimethylate tandem adenosines by PfKsgA1 [144]. The location has been found to reside in the 3′ end of the small subunit stem loop of rRNA that assists its ability to assemble with other necessary components and relocate itself within the cell towards the cytosol and mitochondria [144]. Accumulating evidence indicates that stable RNA structures in mRNAs stall *P*. *falciparum* ribosomes, reducing protein synthesis from translationally active transcripts [145]. Preexisting differences in RNA structure might determine mRNA translation, wherein less structured mRNAs would be more accessible and consequently would be translated more effectively. Alternatively, high translation rates might lead to lower structure in vivo, owing to constant mRNA unfolding by the ribosome. The *P*. *falciparum* transcriptome is highly structured [40,146]. Many highly expressed mRNAs in *P*. *falciparum*, which are essential for parasite development and pathophysiology, have extensively structured coding regions (CDS) [40]. Proteomic characterization of the malaria reader protein complex identified PfYTH.1 in a molecular complex with translation initiation factors raises some interesting possibilities—one is that extensive secondary structure in *P*. *falciparum* mRNAs could provide stability and that engagement of PfYTH.1 to m^6^A expedites recruitment of translational machinery, including ribosomes, which are then responsible for the unfolding of CDS RNA structures by their intrinsic helicase activity during translocation. Alternatively, mRNAs with low structures result in a further decrease of m^6^A enrichment and PfYTH.1 interactions, leading to degradation of mRNAs. These mechanistic possibilities are elaborated in Fig 2. The fingerprint of RNA modifications can lead to unique secondary structures that could aid in the necessary function whether it be RNA-RNA, RNA-DNA, or RNA-protein associated events.

YTHDC2 is an m^6^A reader protein that contains a helicase domain belonging to DExH family proteins, which promotes RNA helicase activity particularly acting in the 3′ to 5′ direction [147]. Through various proteomic assays, it was found to form a complex with XRN1 and MEIOC proteins to successfully alleviate mRNA structure adjacent to m^6^A locations [147]. In *Plasmodium*, PfDDX17 is an ATP-dependent RNA helicase found most abundantly in the trophozoite stage, suggesting its involvement in resolving mRNA structures for modulating protein expression in other asexual, blood stages [148].

It remains ultimately unknown if m^6^A plays a role in resolving secondary structures in *Plasmodium*, *Toxoplasma*, and *Trypanosoma*; however, its enrichment has been found within the coding sequence implying its placement being intentional. Intriguingly, it has been recently demonstrated that *P*. *falciparum* contains more secondary structures (approximately 71.4%) in protein coding mRNAs compared to human, yeast, and metazoan species (approximately 37% to 60%) [40]. The timely expression of virulence-associated proteins in parasitic protists must be regulated in a calculated manner to overcome immunological barriers presented by the host throughout the IDC stages. Therefore, understanding the cooperativity between and RNA structures and m^6^A modifications will be critical as it is known to regulate protein translation in other organisms [7,53,149].

## 7. Advances in molecular technologies for profiling RNA base modifications

m^6^A was first detected in polyadenylated RNAs in the 1970’s [47,150]. However, identification of individual m^6^A-containing RNAs remained a challenge due to a lack of methods for distinguishing m^6^A from unmodified A. Unlike other RNA modifications, the m^6^A modification does not interfere with nominal RNA base pairing properties, making it difficult to detect through traditional sequencing techniques [151,152]. Most strategies for global m^6^A mapping have relied on immunoprecipitation of methylated RNA using m^6^A-detecting antibodies. These include MeRIP-Seq [68], m^6^A-Seq [48], and m^6^A-CLIP [153]. However, these methods suffer from high input requirements and lack of antibody specificity and sensitivity. More recently, several antibody-free methods have been developed, such as m^6^A-REF-Seq [154], MAZTER-seq, m^6^A SAC-seq, and DART-Seq [155]. These approaches have enabled nucleotide-resolution m^6^A mapping from low amounts of RNA and even in single cells [156] and they have provided much-needed tools for not only identifying m^6^A sites but also quantifying m^6^A stoichiometry.

### 7.1 Mapping of m^6^A-methylated RNAs by immunoprecipitation and mass spectrometry

A key bottleneck in the detection and analysis of m^6^A and other modifications is the availability of sensitive, quantitative, and high-throughput techniques to survey modifications transcriptome-wide. RNA contains about 7 times the amount of methyl modifications compared to those found on DNA [157]; however, only recently more accurate, nucleotide resolution detection strategies for m^6^A and other methyl modifications have been developed [155]. MeRIP-seq was the first method for global m^6^A detection and uses m^6^A antibodies to immunoprecipitate methylated RNAs [68,158]. However, this method has a limited resolution of approximately 100 to 200 nucleotides [158,159]. Thus, it can be difficult to determine whether a methylated region contains a single m^6^A site or multiple sites [70,159]. Additionally, the RNA yield ranges from 1% to 10%, requiring a large amount of starting material to have sufficient RNA for sequencing [158,159]. Although this is true, isolating m^6^A-enriched transcripts has many alternative applications [158]. However, miCLIP utilizes this antibody-based approach to achieve nucleotide resolution targets when in conjunction with crosslinking to induce mutational signatures for detection via sequencing [160].

LC-MS/MS was among the first strategies to shed light on the abundance of m^6^A in *P*. *falciparum*’s asexual developmental stages in human RBCs [70], in addition to other RNA modifications. There are many advantages to using this technique, which include the selectivity and specificity. Mass spec for proteomic identification is very well known that makes this application interesting when intended for nucleotide modification identification. The components are predominantly the same, which is creating an ion source, analyzation, fragmentation, and detection. Each of the peaks on the ion chromatogram represents concentration differences between alternative modifications. A full spectra quantification can then be utilized to analyze particularly m^6^A using C12 and C13 isotopes [70] using *Escherichia coli*, or other prokaryotic species, as a viable candidate to act as a normalizing control.

**Fig 3:**
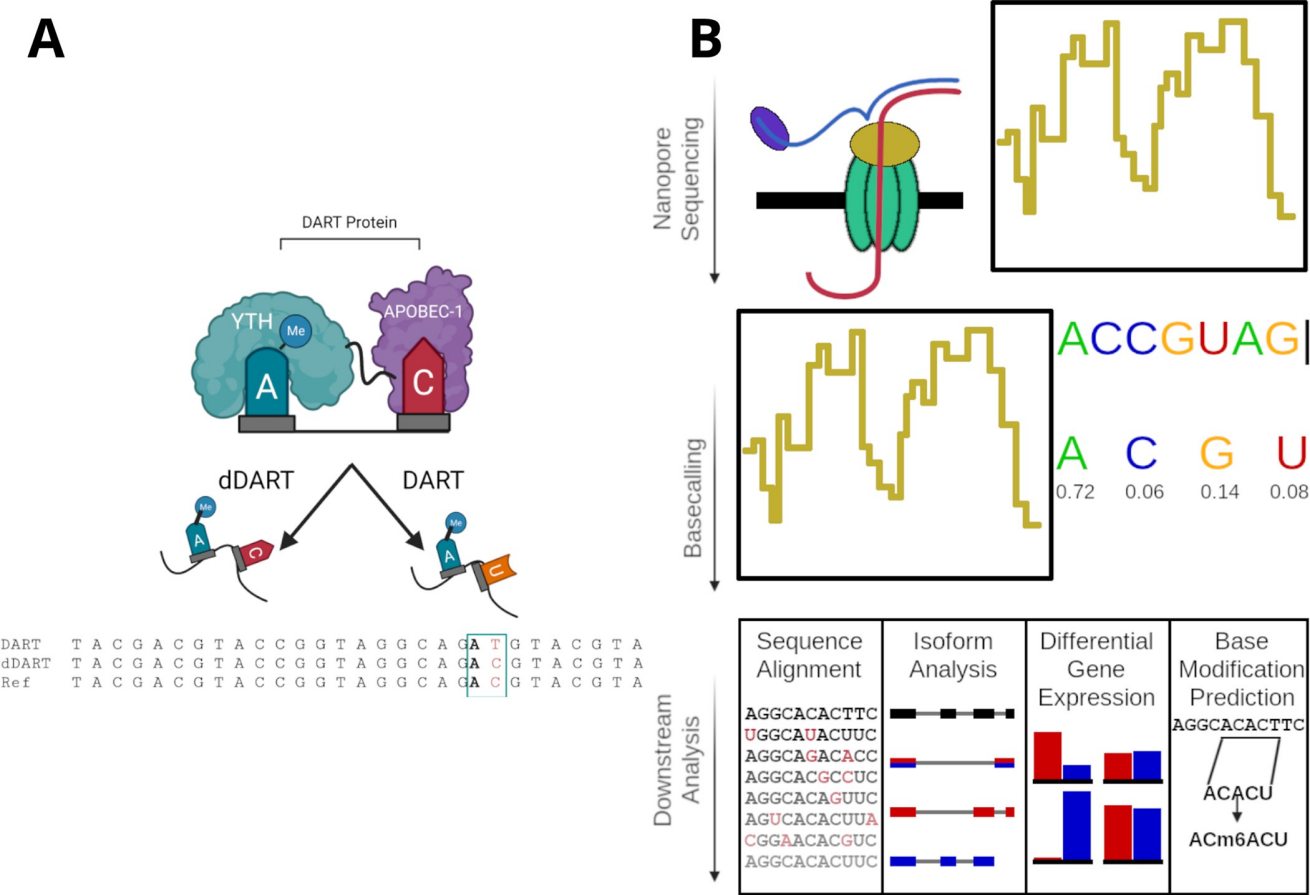
**(A)** Schematic representing m^6^A detection by DART-seq. Targeted deamination to detect m^6^A is achieved by using an RNA editing enzyme, APOBEC1, which is fused to the YTH domain of m^6^A reader protein to guide C-to-U editing at cytidine residues adjacent to m^6^A sites. The mutation achieved by YTH-APOBEC-1 deaminase in the location +1 of m^6^A. dDART has a mutated YTH docking site and therefore samples treated with this enzyme serves as a negative control. **(B)** Schematic of Oxford Nanopore Sequencing (ONS).

In the last few years, several alternative methods have been developed for mapping m^6^A without the need for antibodies. For example, the DART-seq method uses a fusion protein consisting of the YTH domain tethered to the cytidine deaminase, APOBEC1, to guide C to U editing at cytidines that invariably follow m^6^A sites [8] (Fig 3) [155]. DART-seq provides several advantages over conventional antibody-based m^6^A mapping methods. First, since the YTH domain does not recognize m^6^A_m_ (ref), DART-seq enables selective identification of m^6^A and overcomes the issues of cross-reactivity that challenge antibody-based approaches. Second, antibody-based methods require a large amount of input RNA, which could be a major limiting factor for hard-to-grow cells in large quantities, particularly in the case of *P*. *falciparum*. However, DART-seq can be used in vitro to profile m^6^A using as little as 30 nanograms of total RNA and has recently been used to map m^6^A in single cells [156,161]. Therefore, DART-seq enables global m^6^A profiling from limited quantities and is therefore a particularly attractive approach for studies involving *P*. *falciparum*.

### 7.2 Mapping of m^6^A by long-read direct RNA sequencing (DRS)

Oxford Nanopore Technologies (ONT) has arisen as a third/fourth generation sequencing technology capable of directly sequencing DNA and RNA samples without the need of cDNA conversion. Recently, direct RNA sequencing using ONT sequencers have been used to detect m^6^A and other RNA modifications [55,56]. In traditional ONT analyses, total RNA is extracted from the biological sample. Poly(A) mRNA are then enriched from the sample, typically through the use of microbeads or other chromatographic methods [57,58]. A corresponding cDNA is often synthesized to prevent the RNA from forming secondary structures and to protect against degradation from RNases. An RNA sequencing adaptor (RMX), carrying a “motor protein” is ligated to the 3′ end of the RNA, while a “tether” protein is ligated to the 5′ end of the cDNA.

The extracted cDNA molecules are injected onto a flow cell channel connected to an ONT reader device. The flow cell contains hundreds of small nanopore proteins (green), with a nearly 1 nm aperture, embedded in an electro-resistant membrane (black). When the motor protein (yellow) on the cDNA (blue) comes into contact with the nanopore, the difference pulls the connected RNA (red) through the pore, while the tether protein (violet) blocks the cDNA from entering. As the molecule traverses the roughly 5 nucleotide length channel, it disrupts the electrical differential of the membrane. This disruption is captured by a small electrode near the base of the pore, and transmits the change in current to the receiver in the sequencing device. This process continues until the RNA has completely passed through the pore, then the motor protein dissociates, allowing another motor protein to attach and begin the process anew.

The current recorded by the electrode is recorded as a squiggle. Each change in potential is characteristic of a particular 5-mer, the 5 nucleotides contained in the pore, and can be analyzed using machine learning models. This process of reading a squiggle to predict the nucleotide sequence is called base-calling. After base-calling, the resulting long-reads can be used for numerous downstream analyses: de novo genome assembly, transcript isoform discovery, differential gene/transcript expression analysis, or mapping nucleotide modifications at single nucleotide and single molecule resolution [59–61].

Since the development of nanopore sequencing numerous different base-callers have been created, not only to predict the 4 constituent bases, but also to predict specific base modifications. This is possible due to the distinctly different change in current caused by a 5-mer containing a modified base, relative to a 5-mer with an unmodified base. The characteristic difference in current due to RNA base modifications results in base-call errors in those regions, leading to a preponderance of base-call errors at loci encoding for modified bases [62]. Since ONT platform does not require PCR amplification steps, they are supposed to exclude the sequencing or base-calling bias that may arise from GC- or AT-rich genomes [63], such as in *P*. *falciparum* (approximately 90% AT-richness in the intergenic regions).

Researchers have increasingly turned to direct RNA sequencing using the PacBio and Oxford Nanopore platforms as an alternative to these approaches [162–164]. Although further developments are needed to optimize m^6^A identification and increase throughput, these strategies are exciting new additions to the m^6^A detection toolkit. Direct RNA sequencing gives rise to higher read quality by avoiding polymerase slippage in AT dinucleotide repeats, highlighting its importance in analyzing data obtained from unusual genomes of parasitic protists [165]. Additionally, over 50% of *Plasmodium* genes contain introns yet the full characterization of splicing variants remains unknown [166]. Through this technique, precise splicing through unique, stage-specific pre-mRNA processing can be explored that provides greater validation to previous findings as well as novel isoform identification. Most notably, structural variants are abundantly found in the polymorphic var genes that encode for *Plasmodium falciparum* erythrocyte membrane protein-1 (PfEMP1) [167]. These hypervariable sequences are typically located in subtelomeric and intergenic regions resulting in the potential for approximately 60 alternative polypeptide sequences. Expression of these variable antigens can create antigenic diversity which would render multitude of immune evasion strategies led by the parasite. Long-read RNA sequencing by ONT platform, for example, can allow simultaneous detection of the RNA modifications and variations in transcript isoforms, revealing its expression within the timely events of the IDC in hopes for deeper understanding of the posttranscriptional regulation of virulence proteins.

For detection of RNA modifications using PacBio, kinetic changes of reverse transcription reaction should be recorded at the time when the enzyme encounters a modified RNA nucleotide. However, RNA modifications can be identified in its native RNA using nanopore technology by measuring the disruption in current intensity when RNA molecules pass through the nanopores that are embedded in a membrane. In general, one of the strengths of using nanopore technology over PacBio is that of the high sequencing throughput that can be achieved using this technology. While nanopore sequencing provides significantly longer single read fragments than traditional sequencing technologies, it is not without its own shortcomings. Systemic errors in the base-calling process result in a significantly higher error rate (approximately 5%) than other sequencing platforms [168,169]. Additionally, most base-callers only consider the 4 unmodified bases (A, C, G, U), causing clusters of base call errors at and around modified bases [170,171]. Many tools have been developed for identifying RNA modifications in various genomes including pseudouridine, inosine, 5-methyl cysteine (m^5^C), and m^6^A [172–174]. In the case of m^6^A, many different tools have been developed to identify the modification using nanopore data (Table 1). Despite these various tools sharing the same goal of identifying m^6^A modifications using nanopore signal, they vary significantly in their scope and methodology.

**Table 1:** Bioinformatics tools for m^6^A detection based on nanopore sequencing data.

Name	Type of model	Input	Output	Accuracy	Type of cell line
MINES	Random Forest Classifier	bed, bedgraph, fasta	Base resolution of m^6^A locations	Approximately 80%	Human
Nanom^6^A	XGBoost	fast5, fastq, fasta	Base resolution of m^6^A locations	Approximately 90%	Plants (*P*. *trichocarpa* and *Arabidopsis thaliana)* Human
EpiNano	Support Vector Machines	bam, eventalign, fasta	Base resolution of m^6^A locations	Approximately 90%	*S*. *cerevisiae*
m^6^Anet	MIL-based neural network	eventalign	Base resolution of m^6^A location probabilities	Approximately 80%	Human

One of the first tools capable of utilizing nanopore signal for m^6^A prediction at a single nucleotide resolution was MINES, or m^6^A Identification using nanopore sequencing [162] (Table 1). MINES was developed by extracting RRACH motifs within direct RNA sequencing data from HEK293T and HeLa cells. Signals from those RRACH motifs were then compared with m^6^A CLIP data to determine true positives and negatives. The researchers then developed an individual Random Forest Classifier for each of the 4 most abundantly modified A-mers, 5-mers with an A in the central position, in their training set, reporting approximately 80% accuracy. EpiNano uses a significantly different approach, with the developers creating 4 distinct “curlcake” plasmids containing all 1,024 k-mers containing a modified A, while minimizing any potential RNA secondary structure [175]. Using the squiggle data and base-call errors from these curlcakes for training data, EpiNano uses a Support Vector Machines (SVM) architecture to predict m^6^A modifications in external samples. Building upon the approach used by EpiNano, Nanom^6^A uses the same curlcake data and expanded their number of base-call features to include match, mismatch, insertion, and deletion events [164]. In addition to the expanded base-call event features, Nanom^6^A also changes to an Extreme Gradient Boosting (XGBoost) ensemble algorithm and was validated using meRIP-Seq and m^6^A-seq data from human and Populus trichocarpa (Black Cottonwood) samples. M^6^Anet employs an entirely different approach, by using a Multiple Instance Learning neural network and considering squiggle features for not only the target A-mer but also flanking k-mers from various human cell line samples [176].

Despite the improvements in scope and sophistication of the employed methods, existing tools still have significant shortcomings. Some methods like Nanom^6^A and EpiNano, have significant deficiencies in their training data selection, specifically since their synthetic constructs were manufactured using in vitro transcribed sequences, the reference A-mers containing multiple A’s contain multiple modifications (instead of AGACU, m^6^AGm^6^ACU), which are unlikely to occur naturally. As the premise of determining m^6^A modifications using nanopore sequencing is based upon characteristic differences between A and m^6^A, using additionally modified A-mers as the reference to determine the presence of m^6^A in a different context is an obvious source of error and uncertainty. Additionally, each of these methods depends on additional downstream analyses, including base-calling, alignment, and base-correction to perform modification prediction. The reliance on these types of data significantly reduces both the ease of use and specificity of these tools. More specifically, while m^6^ANano demonstrated some ability for transferred learning, with similar performance on both human and *Populus trichocarpa* samples, it is unclear if the other models are capable of such feats, and what the limitations of these tools are for other genomes, especially those with significant differences in both composition (AT richness) and levels of RNA modification like *P*. *falciparum* and other parasites.

Other tools [62,177,178] have been developed to predict modifications from only the raw signal but have been significantly more limited in accuracy. Taken together, it becomes apparent that while these techniques have made significant improvements in a relatively short amount of time, the theoretical “gold standard model,” capable of translating direct RNA sequencing squiggle data from any general species into a sequence of modified and unmodified nucleotide bases remains elusive. However, nanopore direct RNA sequencing technologies remain the most likely source for fast, accurate, and high-resolution base modification predictions for parasite epitranscriptomes.

## Future directions

The dynamic role m^6^A has proven its significance depending on its location along the transcript, the unique recognition by RBPs, and crosstalk from other intrinsic features such as secondary structure which has justified the continuation of investigation. Highly AT-rich genomes, such as *P*. *falciparum*, have adapted interesting methods of posttranscriptional regulation involving these m^6^A mediated events that the molecular parasitology field has not yet fully uncovered, and many questions are left to be currently answered. Emerging studies identified several core enzymes that are involved in RNA epigenetic modifications, much as methylation and pseudouridylation in parasitic protists (Fig 1A). Recent sequence-based epitranscriptomic mappings have provided evidence of stage-specific parasite RNA reprogramming via dynamic modifications of mRNAs and tRNAs and also pointed out key regulators in this process, such as the “reader” proteins. Emerging studies have now demonstrated a comprehensible role for the parasite epitranscriptome in regulating mRNA stability via polyadenylation process due to fact that polyadenylation-specificity factor itself harbored m^6^A reader domain in apicomplexan parasites. A new study in the *Trypanosoma* parasite uniquely showed that m^6^A positively regulates mRNA stability. Additionally, the recent study on tRNA epitranscriptome unearthed the importance of these modifications in codon bias and protein translation in parasites, which could be a newer paradigm for gene regulation in these infectious agents. Although some research has only scratched the surface and others have provided definitive proof of the importance of this work through unique methods, the exciting fact is the field is heading to understand more about epitranscriptomic regulation in human parasitic species important in translational regulation of parasite-specific proteins. On the therapeutic side, specific details about m^6^A mediated 3′ end processing in all species that express the YTH containing polyadenylation ortholog should be exposed to provide clues for targeting “plant-like” polyadenylation factors in parasites. Splicing variants exist in most eukaryotic species and diligent work has begun to address alternative splicing associated with m^6^A in mammals, however, groundwork needs to be done in protozoans to create a similar foundation of knowledge.

There are significant mechanistic details that remain to be discovered in the field of epitranscriptomics. Investigations into the factors that determine which readers are recruited to m^6^A to control mRNA fate will be important for our understanding how m^6^A contributes to cellular function in diverse cell types. Notably, studying the crosstalk within the mRNA dynamics has provided the need for the long-read methods and direct RNA-sequencing techniques that are important for the field to advance. However, the development of new, quantitative and qualitative methods for m^6^A detection has enabled new opportunities for researchers to study m^6^A in diverse organisms and to better understand how m^6^A abundance varies across individual sites and RNAs [179]. The dependency on protein fluctuation during the asexual stages of *P*. *falciparum’s* life cycle has created difficulties in determining precise expression when utilizing short-read sequencing instruments. Direct RNA sequencing allowing long-read capabilities has opened the door for deciphering transcript levels from characteristic repeat sequences and splicing variants during its transcriptome-wide data mining potential.

**Fig 4:**
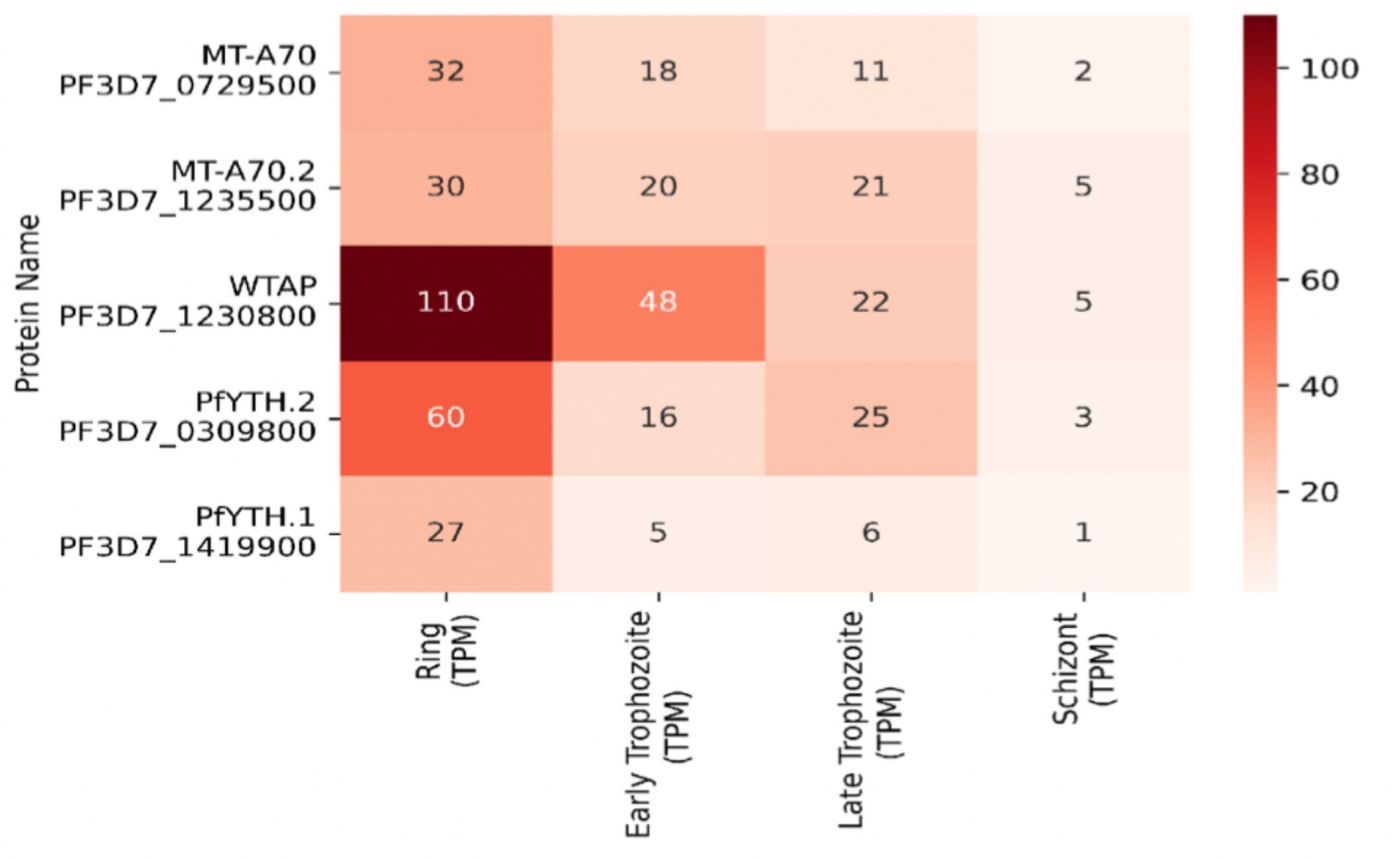
Heat map representation of transcript abundance of the m^6^A writer and reader proteins, MT-A70, MT-A70.2, WTAP, PfYTH.2 and PfYTH.1 in *P*. *falciparum* asexual RBC cycle derived from transcriptomic data (Plasmodb.org) [184]. TPM, transcripts per million.

As seen in Fig 4, there is obvious coordination of m^6^A-associated proteins in *P*. *falciparum* in the context of life cycle inside of human RBCs that provides greater insight into timeliness of mRNA processing events. Exploration of stage-specific, global transcript levels correlating to reader protein abundance can help indicate individual reader roles relating to the transcripts’ fate. Further guidance and understanding of how the epitranscriptome is utilized can allow the molecular parasitology field to expand on spatiotemporal requirements to gain insight on metabolomics, immune evasion, transmission, RBC invasion, cell cycle regulation, etc., ultimately to understanding pathogenesis in a more dynamic, RNA-inclusive manner.
